# Cats are more susceptible to the prevalent H3 subtype influenza viruses than dogs

**DOI:** 10.1080/21505594.2025.2605799

**Published:** 2025-12-17

**Authors:** Jie Deng, Chunhui Ma, Junting Yu, Bo Chen, Shoujun Li, Pei Zhou

**Affiliations:** Guangdong Technological Engineering Research Center for Pets, College of Veterinary Medicine, South China Agricultural University, Guangzhou, Guangdong, People’s Republic of China

**Keywords:** Influenza a viruses, dogs, cats, intermediate host, infection

## Abstract

Recent reports have highlighted the increasing frequency of influenza A virus (IAV) spillover events from other species to dogs and cats. IAV, particularly the H3 subtype, exhibits a broad host range and a propensity for interspecies transmission, as exemplified by the sustained circulation of H3N2 and H3N8 canine influenza viruses in dog populations. This raises concerns about the potential role of companion animals as intermediate hosts in influenza virus transmission. To evaluate the susceptibility of dogs and cats to the prevalent H3 subtype influenza viruses, we experimentally inoculated groups of both species with three prevalent influenza viruses: H3N2 avian influenza virus (AIV), H3N8 avian influenza virus, and H3N2 swine influenza virus (SIV). Results showed that while all inoculated dogs exhibited seroconversion to all three viruses at 7, 14, and 21 days post-inoculation (dpi), they displayed no clinical signs, viral shedding, or evidence of viral replication in their organ tissues. In contrast, despite the cats did not exhibit apparent clinical signs, all inoculated cats exhibited seroconversion to all viruses at 7, 14 and 21 dpi, sustained nasal viral shedding for approximately one week, and demonstrated viral replication in their lungs, trachea, and nasal turbinate. Our findings underscore the higher susceptibility of cats compared to dogs to H3 subtype influenza viruses. These results emphasize the critical need for enhanced surveillance of cats within the influenza virus transmission network.

## Introduction

Influenza A virus (IAV) presents a significant threat to both human and animal health. Through years of genetic variation and interspecies transmission, IAV has evolved to infect a wide range of hosts, including humans, birds, pigs, horses, dogs, and cats [1]. This evolution is primarily driven by viral mutations [2] and reassortment events that occur when a host is co-infected with two distinct IAV strains [3,4]. Pigs, often described as “mixing vessels” for influenza viruses [5], have been reported in numerous human/avian/swine influenza virus reassortment events [6,7], causing the 2009 H1N1 pandemic [8]. Similar to pigs, dogs and cats possess both SAα-2,3 Gal (avian influenza virus receptors) and SAα-2,6 Gal (human influenza virus receptors) in their respiratory tracts [9,10]. Previous experimental infection has shown that cats and dogs are susceptible to human seasonal H3N2 influenza virus [11] [12]. This raises concerns about their potential role as intermediate hosts in IAV transmission.

**Figure 1.**
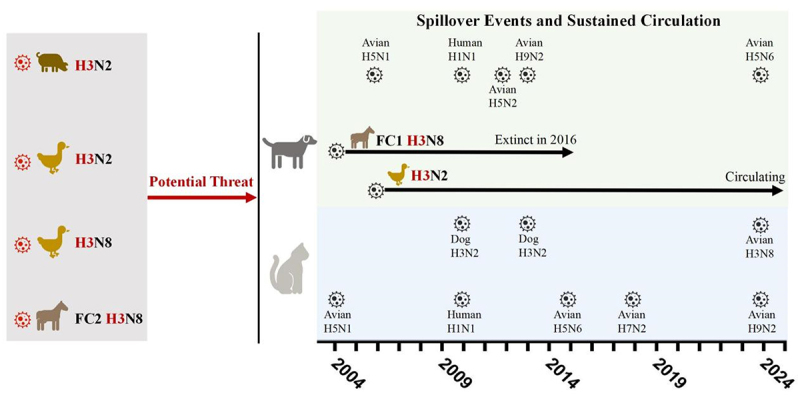
The sustained circulation, potential threat and spillover infections of IAV in dogs and cats in the past 20 years. Spiky ball represents avian influenza viruses from other species that have sporadically spilled over to infect dogs or cats. Solid arrow represents that the virus strain has established sustained circulation.

H3N8 CIV and H3N2 CIV are the predominant IAV strains circulating in dogs. H3N8 CIV originated from equine influenza virus (EIV) H3N8 and was first reported in Florida in 2004 [13,14], while H3N2 CIV originated from avian influenza viruses and was first isolated in 2006 [15]. In contrast, while stable influenza virus circulation has not been established in cats, H3N2 CIV has been shown to infect cats and has natural feline outbreaks in shelters[16] . Sporadic cases of IAV spillover infections have been reported in dogs and cats (Figure 1). Notably, an avian-origin H7N2 AIV efficiently spread among cats in multiple US shelters during 2016–2017, which demonstrates the capacity of cat-to-cat transmission. Although most of these spillover events have not led to widespread outbreaks, they demonstrate the susceptibility of these companion animals to various IAV strains. This highlights the potential for dogs and cats to facilitate IAV reassortment and transmission, posing a potential risk to public health. However, our understanding of the infectivity and pathogenicity of different H3 subtype IAVs in dogs and cats remains limited, with a significant dearth of experimental data.

H3 subtype influenza viruses have a broad host range and generally low pathogenicity. H3N2 and H3N8 are frequently detected in birds and canines [17]. Studies have demonstrated that H3 subtype AIV can undergo reassortment, potentially altering their pathogenicity and enabling them to overcome species barriers and infect humans [18]. The 1968 Hong Kong H3N2 influenza pandemic serves as a prime example, resulting from the reassortment between human H2N2 and avian H3 IAVs [19]. In April 2022, China reported the first human case of H3N8 AIV infection in Henan Province, followed by other cases in Hunan province [20–23]. Sequence analysis revealed that the isolated viruses shared the highest homology with avian H3N8 viruses, suggesting sporadic interspecies transmission from birds to humans [20,23]. Notably, in the epidemiological investigation of the first case, close contact with pet dogs and cats was reported in addition to contact with free-range poultry. However, the role of dogs and cats as potential intermediate hosts in these transmission events remains unclear.

This study aims to investigate the susceptibility of dogs and cats to prevalent H3 subtype IAVs, including H3N2 AIV, H3N8 AIV, and H3N2 SIV (Figure 1). By conducting experimental infections in dogs and cats and monitoring clinical signs, virus shedding, pathological changes, and antibody responses, we aim to study the infection characteristics of H3 subtype IAVs in these companion animals.

## Materials and methods

### Viruses

The following influenza viral strains were used in this study: H3N2 AIV (A/duck/Guangdong/W12/2011) (Accession Number: JX175250.1); H3N8 AIV (A/Gallinula/Guangzhou/A1/2017) (Accession Number: ON287054.1); H3N2 SIV (A/Swine/Guangdong/FS4/2015) (GISAID isolate-ID: EPI_ISL_249845); H3N2 CIV (A/canine/Guangdong/1/2006) (Accession Number: GU433351.1). The viral titers were evaluated by EID_50_/ml assay. Virus stocks were propagated in 9- to 12-day-old embryonated specific pathogen-free (SPF) chicken eggs and titrated using the EID_50_ assay.

### Animals and grouping

Twenty 9- to 11-week-old beagles and twenty 9- to 12-week-old domestic shorthair cats were obtained from the Guangdong Yunfu Canine and Feline Scientific Research Center (Ramical), all seronegative for influenza A viruses, were used in this study. Animals were randomly divided into five groups for both beagles and shorthair cats separately: three experimental groups, one positive group (H3N2 CIV inoculation) and one negative group. Each experimental group consisted of four animals. Each group of animals was housed in separate cages. All animals were anesthetized with propofol (1–2.5 mg/kg) and intranasally inoculated with 10^6^ EID_50_ of the corresponding virus in 1.0 mL PBS. Control groups were inoculated with 1.0 mL pathogen-free SPF chicken embryo allantoic liquid.

### Clinical signs and seroconversion

Clinical signs and rectal temperature were monitored daily for 14 days post-inoculation (dpi). Nasal swabs were collected daily from 1 to 14 dpi and titrated by EID_50_ assay in SPF chicken eggs. Blood samples were collected at 0, 3, 5, 7, 14, and 21 dpi for serological antibodies assessment, treated with receptor-destroying enzyme (RDE), and subjected to hemagglutination inhibition (HI) assay.

### Viral replication and pathological examination

At 4 dpi, one animal from each group was euthanized with an intravenous injection of pentobarbital sodium (150–200 mg/kg). Lung, trachea, nasal turbinate, heart, liver, spleen, kidney, intestine, stomach, and brain tissues were collected. In consideration of animal welfare, euthanasia was performed on only one animal per group in this study, and three tissue samples were collected from each type of tissue. Tissues were fixed in 10% neutral buffered formalin, processed for hematoxylin and eosin (H&E) staining, and immunohistochemistry (IHC) using Anti-Influenza Virus NS1A Binding Protein by ServiceBio. All tissues were weighed and homogenized in 1 mL of PBS per gram, then centrifuged to obtain the supernatant, which was titrated using the EID_50_ assay to assess viral replication.

### Statistical analyses

All viruses sequences used for phylogenetic analysis were downloaded from NCBI (https://www.ncbi.nlm.nih.gov/) and GISAID (https://gisaid.org/) based on the following criteria: (1) sequences from diverse host species (e.g. avian, canine, feline, human, swine, equine) to represent the broad host range of H3 viruses; (2) sequences isolated between 2000 and 2023 to focus on contemporary strains. Align the gene fragments of the reference strain and isolates using Mafft 7.058 software, and remove the repetitive sequences in the gene fragments using PhyloSuite; Sequence alignment was performed using Mega X 7.0.26 software; Phylogenetic analysis was conducted using IQ-TREE; Draw the phylogenetic tree using Itol. All data measurements were performed in triplicate. Data were analyzed by two-way ANOVA test using GraphPad Prism, version 8.

### Guidelines statement

This study has been conducted with rigorous compliance to the ARRIVE guidelines.

## Results

### Phylogenetic analysis

**Figure 2.**
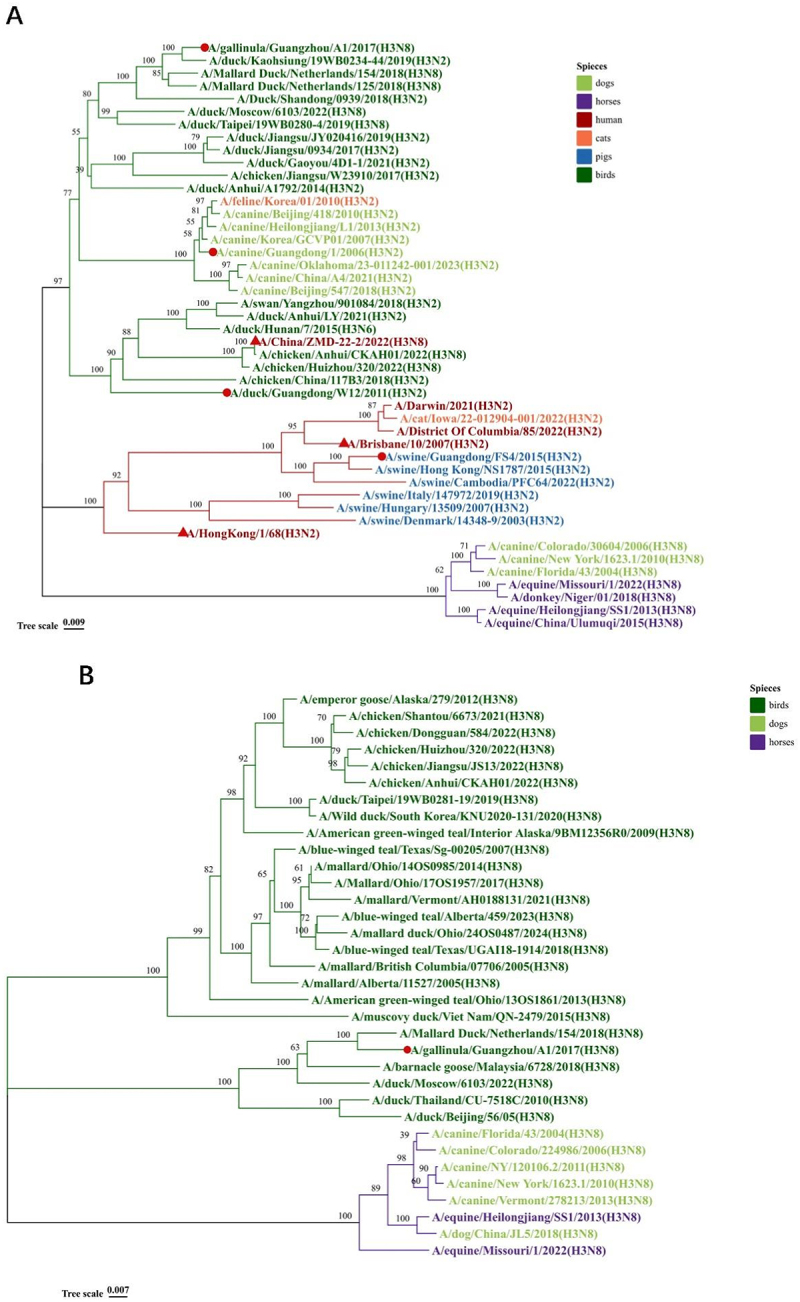
Phylogenetic tree of the ha genes of 46 H3 subtype IAVs (a) and na genes of 38 N8 subtype IAVs (b). The green branches of the phylogenetic tree represent the Eurasian avian lineage, the red branches represent the human/swine lineages, and the purple branches represent equine lineages. Second, the leaves of the phylogenetic tree are dark green for birds, light green for dogs, red for human, orange for cats, blue for pigs and purple for horses. Thirdly, the viruses used in this study are marked with red circles, and human influenza viruses that have shown to infect dogs/cats are marked with red triangles.

HA gene and NA gene sequences of prevalent H3 subtype IAVs were downloaded from NCBI and GISAID for phylogenetic analysis. Analysis result of HA gene showed that contemporary H3 subtype IAVs can be categorized into: avian lineage, dog/cat lineage, human/swine lineage, and equine lineage (Figure 2(A)). Notably, H3N2 CIV clusters within the avian lineage, while H3N8 CIV belongs to the equine lineage. We selected the earliest isolated representative strain of H3N2 CIV for the positive control in this study. The three experimental strains, H3N2 AIV, H3N8 AIV, and H3N2 SIV were isolated and preserved by our laboratory from samples collected in Guangdong Province, China. The comparative analysis of the NA gene between prevalent H3N8 CIV and H3N8 AIV showed that these viruses cluster on distinct branches within the phylogenetic tree, indicating significant evolutionary divergence (Figure 2(B)). In contrast, H3N8 CIV exhibits closer phylogenetic proximity to H3N8 EIV, consistent with findings reported in previous studies (Figure 2(B)).

### Clinical signs and seroconversion

**Figure 3.**
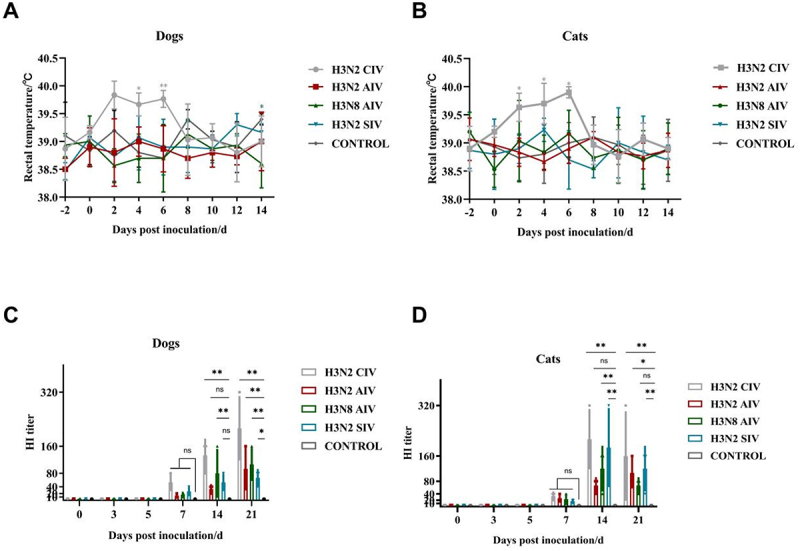
Daily rectal body temperature in dogs (a) and in cats (b) inoculated with H3N2 civ, H3N2 AIV, H3N8 AIV, H3N2 SIV and negative control group. Serological antibodies were detected at 0, 3, 5, 7, 14, 21 dpi in dogs (c) and in cats (d) inoculated with H3N2 civ, H3N2 AIV, H3N8 AIV, H3N2 SIV and negative control group. Differences (*p < 0.05, **p < 0.01, ***p < 0.001) between the experimental and control groups are noted.

Dogs in the H3N2 CIV group displayed clinical signs including sneezing, nasal discharge, and fever (rectal temperature > 39.6°C), while the other four groups of dogs remained clinically healthy (Figure 3). Similarly, cats infected with H3N2 CIV exhibited clinical signs such as sneezing, nasal discharge, and fever between 2 to 6 dpi. In contrast, cats in the H3N2

All dogs inoculated with H3N2 CIV, H3N2 AIV, H3N8 AIV and H3N2 SIV showed seroconversion starting from 7 dpi (Figure 3). HI titers increased over time, reaching peak titers of 213, 93, 106 and 66, respectively, at 21 dpi. Notably, dogs in the H3N2 CIV group exhibited the highest antibody titers among all groups. No seroconversion was observed in the negative control group. Cats inoculated with the respective viruses also seroconverted from 7 dpi. Peak HI titers were observed at 14 dpi in cats inoculated with H3N2 CIV, H3N8 AIV, and H3N2 SIV, reaching peak titers of 213, 120, 106 and 186, respectively. Cats in the H3N2 CIV group displayed the highest HI titers among all groups. No seroconversion was found in the negative control group.

Although no clinical signs such as fever, sneezing, or nasal discharge were observed in dogs and cats inoculated with H3N2 CIV, H3N8 AIV, and H3N2 SIV, the presence of seroconversion indicated successful viral inoculation without overt clinical symptoms.

### Virus shedding

**Figure 4.**
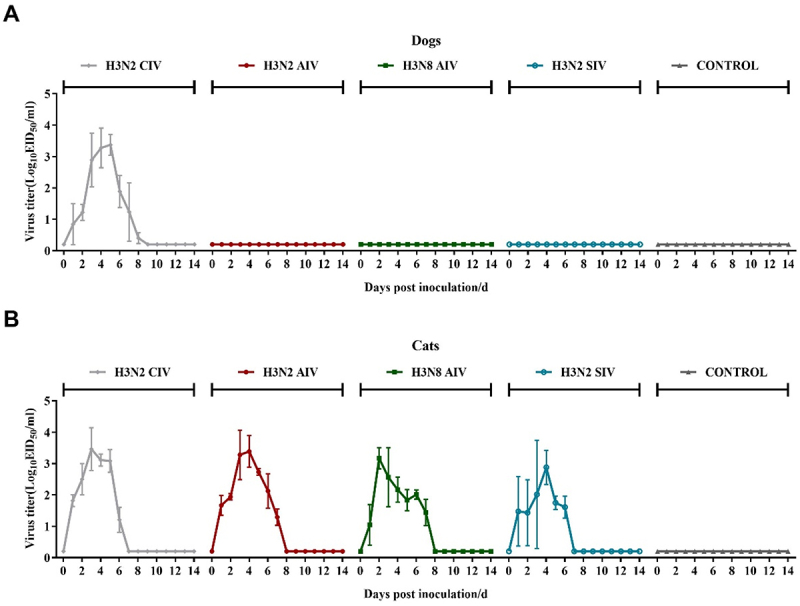
Nasal shedding of viruses in dogs (a) and in cats (b) inoculated with H3N2 civ, H3N2 AIV, H3N8 AIV, H3N2 SIV and negative control group.

In dogs, H3N2 CIV was detected in the nasal swabs of the H3N2 CIV group, with a peak titer of 10^3.17^ EID_50_/mL at 4 dpi (Figure 4). No viral shedding was observed in the other four groups.

Nasal swabs from cats in the H3N2 CIV and H3N2 SIV groups tested positive from 1 to 6 dpi, with peak viral titers reaching 10^3.46^ EID_50_/mL and 10^2.88^ EID_50_/mL at 3 dpi and 4 dpi. Cats in the H3N2 AIV and H3N8 AIV groups also exhibited nasal viral shedding from 1 to 7 dpi, with peak titers of 10^3.38^ EID_50_/mL and 10^3.17^ EID_50_/mL at 4 and 2 dpi, respectively. No viral shedding was detected in the negative control group.

One nasal swab of each positive group was selected to exam viral adaptation mutation. No mutations in HA or NA genes from all selected samples.

### Viral replication

**Figure 5.**
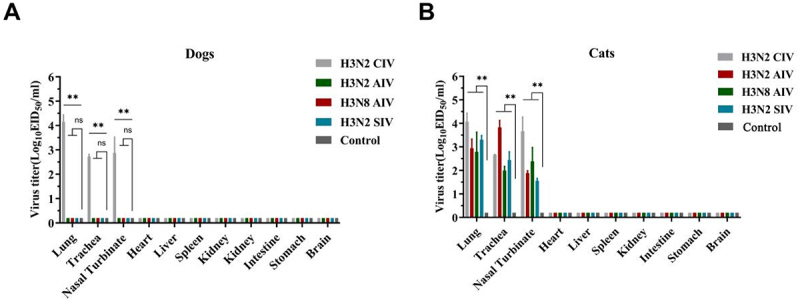
Viral replication at 4dpi in different organs in dogs (a) and in cats (b) inoculated with H3N2 civ, H3N2 AIV, H3N8 AIV, H3N2 SIV and negative control group. Viral titers are expressed as log_10_EID_50_/ml. Differences (*p < 0.05, **p < 0.01, ***p < 0.001) between the experimental and control groups are noted.

At 4 dpi, three tissue samples were collected from each tissue type from one euthanized animal per group to assess viral replication. In dogs, viral replication was observed in the lung, trachea and nasal turbinate of dogs in the H3N2 CIV group. Among these respiratory tissues, the lung displayed the highest viral titer (10^4.15^ EID_50_/mL), followed by the nasal turbinate (10^2.86^ EID_50_/mL) and the trachea (10^2.73^ EID_50_/mL) (Figure 5(A)). No viral replication was detected in the heart, liver, spleen, kidney, intestine, stomach, or brain. Similarly, no viral replication was found in dogs inoculated with H3N2 AIV, H3N8 AIV, H3N2 SIV, or in the negative control group (Figure 5(A)), consistent with the absence of viral shedding in these groups.

In all inoculated groups of cats, viral replication was restricted to the respiratory tract, with no evidence of replication in non-respiratory tissues or in the negative control group (Figure 5(B)). Among the cats inoculated with H3N2 CIV, the lungs exhibited the highest viral titer (10^4.07^ EID_50_/mL), followed by the nasal turbinate (10^3.67^ EID_50_/mL) and the trachea (10^2.68^ EID_50_/mL) (Figure 5(B)). Similarly, in the H3N8 AIV group, the lungs showed the highest viral titer (10^2.79^ EID_50_/mL), followed by the nasal turbinate (10^2.38^ EID_50_/mL) and the trachea (10^2^ EID_50_/mL) (Figure 5(B)). Cats inoculated with the H3N2 SIV also displayed the highest levels of replication in the lungs (10^3.31^ EID_50_/mL), while the viral replication titer in the trachea (10^2.44^ EID_50_/mL) slightly exceeded that in the nasal turbinate (10^1.56^ EID_50_/mL) (Figure 5(B)). In contrast, cats in the H3N2 AIV group exhibited the highest viral titer in the trachea (10^4.38^ EID_50_/mL), surpassing the levels observed in the lungs (10^2.94^ EID_50_/mL) and nasal turbinate (10^1.89^ EID_50_/mL) (Figure 5(B)). The viral replication data were derived from a single animal per group due to ethical constraints. While this might limit statistical inference, the consistency between tissue replication, nasal shedding, and serological response strengthens our observations.

### Pathological changes

**Figure 6.**
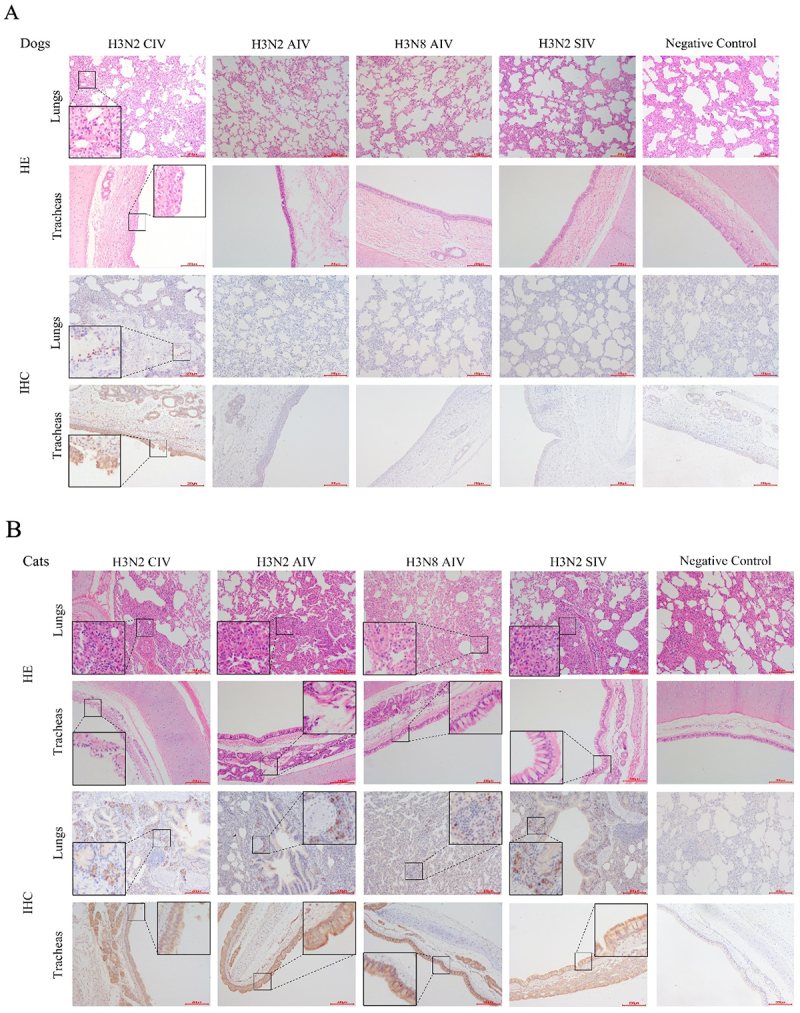
Hematoxylin-eosin (he) and immunohistochemical (IHC) staining images of lungs and tracheas at 4dpi in dogs inoculated with H3N2 civ, H3N2 AIV, H3N8 AIV, H3N2 SIV and negative control group (a), in cats inoculated with H3N2 civ, H3N2 AIV, H3N8 AIV, H3N2 SIV and the negative control group (b). Magnification of the HE and IHC photos were ×100, and the ruler is 200 μm.

Microscopic examination of lungs and tracheas tissues from dogs inoculated with H3N2 CIV revealed lesions characterized by pulmonary interstitial hyperplasia, mainly comprising interstitial cells and inflammatory cells (Figure 6(A)). Immunohistochemistry (IHC) confirmed the presence of viral antigens in these infected tissues (Figure 6(A)). In contrast, no lesions or viral antigens were detected in tissues from dogs in H3N2 AIV, H3N8 AIV, H3N2 SIV groups, or in the negative control group (Figure 6(A)).

In cats inoculated with H3N2 CIV, lungs exhibited severe pulmonary interstitial hyperplasia and inflammatory cell infiltration, and trachea showed necrosis of mucosal epithelial cells (Figure 6(B)). Lungs from cats inoculated with H3N2 AIV displayed significant inflammatory cell infiltration around the outer membrane of the bronchus, primarily consisting of lymphocytes and mononuclear macrophages, accompanied by substantial epithelial cell shedding into the bronchial lumen mixed with inflammatory cells (Figure 6(B)). Lymphocytes and mononuclear macrophages infiltration of varying degrees were also observed in lungs from cats in H3N8 AIV and H3N2 SIV groups (Figure 6(B)). Lungs from cats in the negative group only showed mild pulmonary interstitial hyperplasia. Viral antigens were detected by IHC in the lungs and tracheas of cats inoculated with H3N2 CIV, H3N2 AIV, H3N8 AIV, and H3N2 SIV (Figure 5(B)). No viral antigens were found in the negative control group.

## Discussion

Given the increasing frequency of IAV spillover events to dogs and cats, coupled with the presence of both avian and human influenza virus receptors in their respiratory tracts, there is growing concern that these companion animals may serve as intermediate hosts for influenza viruses. This concern is especially heightened by the increasing number of companion animals, particularly dogs and cats, within human households. Recent reports of sporadic human infections with H3N8 AIV highlight the potential threat posed by H3 subtype influenza viruses [23,24].

To investigate the susceptibility of dogs and cats to prevalent H3 subtype influenza viruses, we experimentally inoculated both species with H3N2 AIV, H3N8 AIV, and H3N2 SIV. Our results demonstrated that dogs were not susceptible to these three H3 subtype influenza viruses, despite seroconversion post-inoculation. While dogs were initially considered relatively resistant to influenza viruses, equine-origin H3N8 CIV was first reported in the United States and has been stably circulating in dog populations since 2004. In 2010, our laboratory reported an avian-origin H3N2 CIV strain isolated in 2006 [15]. Subsequently, H3N2 CIV has spread widely, establishing stable circulation in Asian and North American dog populations [25,26]. However, recent data confirms that H3N8 CIV became extinct globally in late 2016, and H3N2 CIV has died out repeatedly in North America [27], reflecting the lack of sustained viral circulation in canine populations. This epidemiological pattern aligns with our experimental findings of low susceptibility of dogs to these prevalent H3 subtype influenza viruses, although continued surveillance of canine influenza remains crucial.

In contrast to dogs, cats were susceptible to infection with H3N2 AIV, H3N8 AIV, and H3N2 SIV. These viruses replicated in the respiratory tract of infected cats, and continuous nasal viral shedding was observed for several days post-inoculation. Seroconversion was detected at 7, 14 and 21 dpi. Notably, except for the H3N2 CIV positive control group, cats inoculated with other H3 subtype influenza viruses did not exhibit apparent clinical signs such as coughing or fever. Only further histopathological examination revealed lesions. These findings suggest that due to the low pathogenicity of H3 subtype influenza viruses, infected animals may often be asymptomatic or exhibit mild clinical signs, making them difficult to be distinguished and facilitating the continued transmission and evolution of these viruses. While stable influenza virus circulation has not yet been established in cats, their higher susceptibility to spillover infection is generally offset by low onward transmission due to limited cat-to-cat contact at home, except in confined environments such as shelters. Furthermore, H7N2 AIV and H3N2 CIV were found widely spread among cat shelters for a period of time, indicating the potential for efficient influenza virus transmission under such conditions. Previous studies have demonstrated their susceptibility to various animal influenza viruses, including H5N1, H5N6, H7N2, H9N2, as well as human H1N1 influenza virus (Figure 1). The high susceptibility of cats highlights the need for enhanced surveillance of feline influenza viruses to prevent them from becoming the intermediate host or even the next “mixing vessel” for influenza viruses.

Compared to dogs, cats exhibited a higher susceptibility to common H3 subtype influenza viruses. However, the molecular mechanisms underlying this species-specific susceptibility to H3 subtype influenza viruses remain incompletely understood. Emerging evidence suggests that host-virus interactions at multiple levels dictate these differences. Notably, both dogs and cats exhibit similar sialic acid receptor distributions, with SAα2-6 Gal and SAα2-3 Gal co-expressed across multiple tissues [10] Wang et al. [2013]. This shared receptor profile suggests a comparable molecular basis for cross-species susceptibility to H3N2 viruses. However, recent studies highlight that receptor specificity alone may not fully explain interspecies differences. For instance, avian-origin H3N2 CIV strains retain binding affinity for SAα2-3 Gal despite adapting to dogs, implying additional factors like HA glycosylation patterns or novel host receptors (e.g. mGluR2 in endocytosis) may modulate infection dynamics [28]. Second, innate immune evasioncontributes to interspecies variation; for instance, NS1 protein-mediated suppression of RIG-I/TRAF3 signaling may be counter balanced by host factors like hnRNPH1 (Wang et al. 2025), and polymorphisms in ANP32A/B can modulate polymerase activity (e.g. PB2 E627K) across species [29]. Future studies should integrate histopathology with molecular profiling (e.g. cytokine networks, single-cell transcriptomics) to link observed lesions to specific immune and viral mechanisms. In addition, whole-genome sequencing of single-virus particle shed in feline nasal secretions should be performed to identify adaptive mutations that may arise during replication in cats, which would provide critical insights into viral evolution and host adaptation.

Through experimental infection, our study significantly advances our understanding of the infectivity and pathogenicity of various H3 subtype influenza viruses in dogs and cats. Based on our previous studies and the current findings, we can conclude that dogs are resistant to H3N8 EIV2, H3N2 AIV, H3N8 AIV, and H3N2 SIV, while cats are susceptible to H3N8 EIV2, H3N2 AIV, H3N8 AIV, H3N2 SIV, and H3N2 CIV (Table 1). This pronounced susceptibility of cats, however, contrasts with the epidemiological observation that stable influenza virus circulation has become established in dog populations (e.g. equine-origin H3N8 CIV and avian-origin H3N2 CIV) but not in cats. A critical factor explaining this paradox is the demographic behavior of cats. Specifically, efficient cat-to-cat transmission is highly dependent on close contact, a condition typically limited in dispersed household settings but readily met in high-density confined environments such as animal shelters.This demographic constraint is supported by historical evidence of H7N2 and H3N2 influenza outbreaks in feline shelters (Figure 1), underscoring the imperative for targeted surveillance in populations where close proximity may promote viral adaptation and sustained transmission.

**Table 1.** Based on the experimental infection results in this study, the summary of susceptibility of dogs and cats to potential threatening H3 subtype influenza viruses.

	Subtype	Previous Susceptibility	Experimental Infection
Dog	H3N2 AIV	Unknown	Unsusceptible
	H3N2 SIV	Unknown	Unsusceptible
	H3N8 AIV	Unknown	Unsusceptible
	H3N8 EIV (FC 2)	Unsusceptible [30]	Unsusceptible
Cat	H3N2 AIV	Unknown	Susceptible
	H3N2 SIV	Unknown	Susceptible
	H3N8 AIV	Unknown	Susceptible
	H3N8 EIV (FC 2)	Susceptible [31]	Susceptible

## Data Availability

The data that support the findings of this study are openly available in Figshare (The dataset can be accessed by entering the article title on the following link: https://doi.org/10.6084/m9.figshare.28432856) []. All relevant data are within the paper and its Supporting Information files.
